# Zinc Tantalum Oxynitride (ZnTaO_2_N) Photoanode Modified with Cobalt Phosphate Layers for the Photoelectrochemical Oxidation of Alkali Water

**DOI:** 10.3390/nano8010048

**Published:** 2018-01-18

**Authors:** Prabhakarn Arunachalam, Maged N. Shaddad, Mohamed A. Ghanem, Abdullah M. Al-Mayouf, Mark T. Weller

**Affiliations:** 1 Electrochemistry Research Group, Chemistry Department, College of Science, King Saud University, Riyadh 11451, Saudi Arabia; mshadad@ksu.edu.sa (M.N.S.); mghanem@ksu.edu.sa (M.A.G.); amayouf@ksu.edu.sa (A.M.A.-M.); 2 Department of Chemistry, University of Bath, Bath BA2 7AY, UK; m.t.weller@bath.ac.uk

**Keywords:** cobalt phosphate, tantalum oxynitride, water oxidation, photoelectrochemistry, solid-state synthesis

## Abstract

Photoanodes fabricated by the electrophoretic deposition of a thermally prepared zinc tantalum oxynitride (ZnTaO_2_N) catalyst onto indium tin oxide (ITO) substrates show photoactivation for the oxygen evolution reaction (OER) in alkaline solutions. The photoactivity of the OER is further boosted by the photodeposition of cobalt phosphate (CoPi) layers onto the surface of the ZnTaO_2_N photoanodes. Structural, morphological, and photoelectrochemical (PEC) properties of the modified ZnTaO_2_N photoanodes are studied using X-ray diffraction (XRD), scanning electron microscopy (SEM), ultraviolet visible (UV−Vis) diffuse reflectance spectroscopy, and electrochemical techniques. The presence of the CoPi layer significantly improved the PEC performance of water oxidation in an alkaline sulphate solution. The photocurrent-voltage behavior of the CoPi-modified ZnTaO_2_N anodes was improved, with the influence being more prominent at lower oxidation potentials. A stable photocurrent density of about 2.3 mA·cm^−2^ at 1.23 V vs. RHE was attained upon visible light illumination. Relative to the ZnTaO_2_N photoanodes, an almost three-fold photocurrent increase was achieved at the CoPi/ZnTaO_2_N photoelectrode. Perovskite-based oxynitrides are modified using an oxygen-evolution co-catalyst of CoPi, and provide a new dimension for enhancing the photoactivity of oxygen evolution in solar-assisted water-splitting reactions.

## 1. Introduction

The development of active and proficient semiconductor photocatalysts for the direct conversion of solar energy to chemical energy has gained attention in the desire to fulfil future energy and fuel demand [1,2]. The use of solar photocatalytic water splitting to produce H_2_ and O_2_ over a heterogeneous photocatalyst has been studied extensively for several years, and has been found to be a favorable process for clean and renewable hydrogen generation. The heterogeneous photocatalytic process function at a solar-energy conversion efficiency of 10% is theoretically able to generate hydrogen at a rate of $1.63/kg H_2_ [3]. However, the development of a photocatalyst for the oxidation of water to yield oxygen gas remains the main hurdle that needs to be overcome in order to establish a technology that is based on water-splitting. The water oxidation reaction is the most difficult half reaction process as it includes four-electron transfer to generate an oxygen molecule, O_2_; therefore there are significant thermodynamic and kinetic limitations to this process [3]. Several polycrystalline photocatalyst materials have been established for solar-assisted water splitting [1,4]; on the other hand, the materials available for the fabrication of efficient photoelectrodes are still limited. The widely investigated photocatalyst is the TiO_2_ photoelectrode, with a band gap (BG) of 3.0 eV; however, this large band is not suitable for the absorption of visible light, and an external bias is generally required to achieve surface redox reactions [5,6,7,8]. Therefore, for improved photoelectrodes, it is indispensable to narrow the BG of the semiconductors used to expand light absorption into the visible region of the solar spectrum.

Significant attempts have been carried out to develop new types of oxide-based semiconductors [9,10,11,12]. In particular, n-type semiconductor photoanodes, such as Fe_2_O_3_, WO_3_, and BiVO_4_ [13,14,15], have been employed to accumulate the solar visible-light photons in stable and proficient water-splitting reactions. However, Scaife reported that it is inherently harder to progress an oxide-based photocatalyst that has an appropriately negative conduction band (CB) and a narrow BG (i.e., <3 eV) for visible-light absorption due to their higher positive valence band (VB) (at ca. +3.0 V vs. NHE) created by the O 2p orbital [16,17]. Other oxide-based photoelectrodes, such as La-doped NaTaO_3_ [18] and Rh-doped SrTiO_3_ [19], show reasonable quantum efficiencies (QEs) in the presence of UV light, and have been utilized as H_2_ evolution photocatalysts in visible-light irradiation.

Oxynitride-based photoanodes offer an alternative to oxide-based anodes for visible-light absorption to produce H_2_ and O_2_ from water at a stoichiometric ratio [17,20,21,22,23,24,25]. In these materials, atomic nitrogen is introduced into the oxygen sites, and it shifts the VB edge potentials to the more negative region via the hybridization of N 2p and O 2p orbitals. Domen et al. demonstrated TaON/co-catalyst-based photoanodes with an incident photon-to-electron conversion efficiency (IPCE) of 76% at 400 nm for water oxidation at a minimal or no external applied bias [20,21,22,23,24,25,26]. Since then, oxynitrides, such as LaTiO_2_N [22], SrNbO_2_N [27,28], BaNbO_2_N [29], among other materials, have been similarly developed as photoanodes [30,31,32,33,34,35]. Few of these have attained superior IPCEs of the order of magnitude of several tens of percent with an externally applied bias that is less than the thermodynamically mandatory oxidation potential (1.23 V) for water electrolysis. Currently, significant research is underway to develop the smaller band-gap photoanodes with a BG of <2 eV (equivalent to 600 nm in the visible absorption spectrum) to oxidize water with the application of minimal bias [33]. Several such photoanodes have been developed, e.g., BaNbO_2_N [21], CoO*_x_*/LaTiO_2_N [22], and BaZrO_3_/BaTaO_2_N [33], which have been found to utilize the absorption of visible-light in addition to appropriate sacrificial reagents. SrNbO_2_N-based photoanodes (BG < 2 eV), function as water-oxidation catalysts with the support of a co-catalyst in visible-light illuminations [27,28,34]. However, the recombination of the photoexcited carriers and poor photon absorption efficiencies are considered as major drawbacks with these systems, and these attributes need to be improved for application in water-splitting reactions. The incorporation of oxygen-evolution electro-catalysts (OECs) with a photon-absorbing substrate shows it to be a suitable plan to develop the field of photocatalysis chemistry, thus increasing the overall solar conversion efficiency [35]. Recently, the use of cobalt-based materials has also been promising for electrochemical applications [36,37,38]. To this end, the amorphous cobalt-phosphate (Co-Pi) appears to be a promising candidate as a co-catalyst material that possesses the advantages of relatively high elemental earth abundances, a self-healing feature, and functionality under benign conditions [39,40]. Recently, we reported the PEC activity of ZrO_2_- and Fe_2_O_3_- modified BiVO_4_ and CoPi/La(Ta,Nb)O_2_N photoelectrodes for water oxidation in alkaline media [32,41], which achieved up to a 5-fold enhancement in the water-oxidation photocurrent at a lower oxidation potential. In contribution to this research, the present work conveys a significance of engaging a cobalt-based electrocatalyst over tantalum oxynitrides via the electrophoretic deposition route to fabricate the photoanodes. Among the various oxynitrides, ZnTaO_2_N belongs to a LiNbO_3_-type structure, which crystallizes in a high temperature paraelectric phase (HTLN-type) structure. ZnTaO_2_N is a perovskite-type oxynitride containing comparatively earth-abundant metals and it offers a remarkable unique property compared to other available oxynitrides. Our aim in this work is to activate the ZnTaO_2_N catalyst for the water oxidation process by incorporating the oxygen evolution reaction co-catalysts. The loading of the CoPi OER co-catalyst could amplify the charge separation and collection of holes generated at the surface of the photoanodes, subsequently improving photocatalytic performance.

Herein, we describe about the cocatalytic influence as well as the PEC performance of the Co-Pi incorporated ZnTaO_2_N photoanodes. The PEC properties of the ZnTaO_2_N electrodes are enhanced predominantly when modified with the Co-Pi catalyst, and its effect is greatest at a lower potential, while the photocurrent is enriched by a factor of 2.5 times relative to that of a bare electrode. The enhancement of the performance can be credited to an improvement in hole collection and charge-separation efficiency at the surface of the oxynitride photoanodes. The present work also demonstrates a viable way for an improvement in the energy-conversion efficiency by coupling OECs with oxynitride photoanodes.

## 2. Results and Discussion

### 2.1. XRD and DRS Analysis

**Figure 1:**
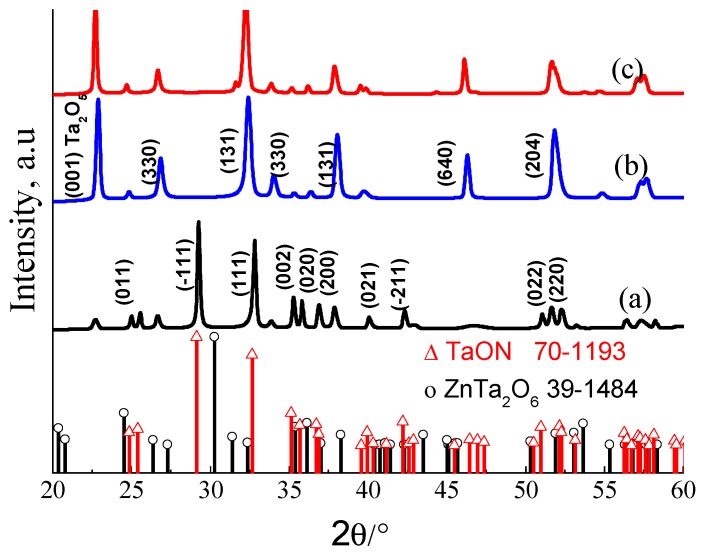
X-ray diffraction (XRD) patterns of (**a**) reference TaON; (**b**) ZnTaO_2_N and (**c**) CoPi/ZnTaO_2_N photo-anodes prepared via electrophoretic and photodeposition.

The ZnTaO_2_N catalyst was prepared using the conventional ammonolysis method, and for comparison, TaON was prepared using a similar procedure. The as-synthesized ZnTaO_2_N powders show an orange-yellow color, while the TaON powder had a yellow color. The electrophoretic deposition (EPD) method was employed to prepare photoanodes of these materials. The average thickness of the ZnTaO_2_N-deposited film on the indium tin oxide (ITO) substrate was measured using a profilometer, and was about 2.5 μm. Figure 1 demonstrates the XRD patterns for the TaON, ZnTaO_2_N, and CoPi/ZnTaO_2_N photoanodes that were fabricated by EPD followed by loading CoPi using photodeposition methods on ITO substrates, and annealing in a nitrogen atmosphere at 450 °C. The XRD pattern of as-synthesized ZnTaO_2_N reveals that all the major peaks can be assigned to a perovskite-type phase that is structurally similar to that of ZnTaO_2_N [28]. The TaON XRD pattern obtained was consistent with the formation of the baddeleyite (monoclinic ZrO_2_) structure. The XRD pattern of TaON photoanodes (Figure 1a) can be well indexed with the phase of TaON (JCPDS # 70-1193). In ZnTaO_2_N films, a small level of peak broadening was observed, which implies a reduction in particle size compared to the TaON photoanodes. The XRD pattern of the electrodeposited ZnTaO_2_N photoanodes (Figure 1b) displays the mixed phase of ZnTa_2_O6 (JCPDS # 39-1484) and TaON.

**Figure 2:**
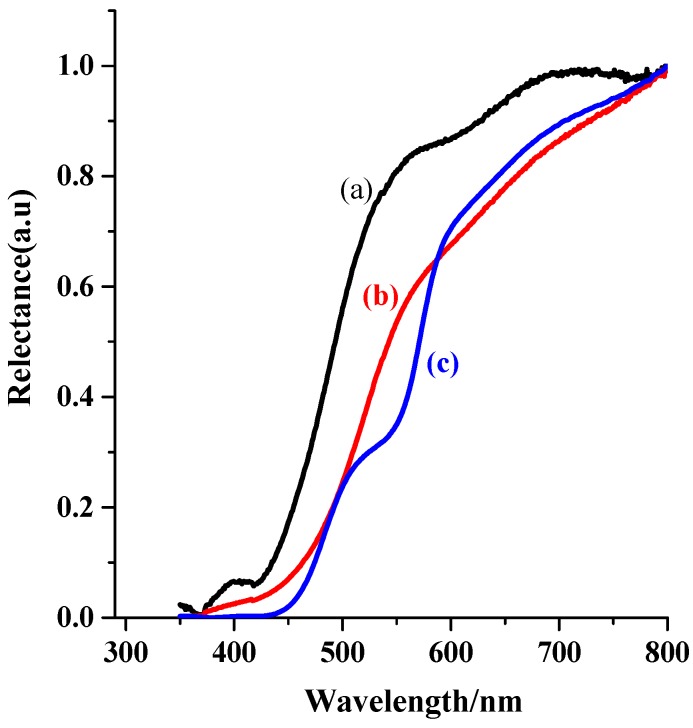
Ultraviolet visible (UV-Vis) reflectance spectra of perovskite-type tantalum oxynitride modified photoanodes of (**a**) TaON; (**b**) ZnTaO_2_N and (**c**) CoPi/ZnTaO_2_N.

The XRD patterns of the CoPi-loaded ZnTaO_2_N photoanodes show that the CoPi deposition did not alter the crystalline phase of ZnTaO_2_N photoanodes, and no XRD peaks corresponding to metal impurities or simple metal oxides were observed. This may be credited to the good dispersion of nanocrystalline CoPi particles over the ZnTaO_2_N. A small shift towards the lower 2θ values was observed in the presence of CoPi-loaded photoanodes, compared with the parent photoanodes, and this was due to a small difference in the sample height in the powder diffractometer. Figure 2 displays diffuse reflectance spectroscopy (DRS) spectra for the various TaON-based photoanodes. All fabricated TaON-based photoanodes have visible light, and the onset of light-absorption features of the band-gap excitation of TaON, ZnTaO_2_N, and CoPi/ZnTaO_2_N was observed at 420, 447, and 452 nm, respectively.

However, CoPi/ZnTaO_2_N photoanodes display dissimilar absorption spectra from that of ZnTaO_2_N, and the absorption edge point is mainly lifted to the higher wavelengths compared with TaON. The BG of the fabricated TaON-based photoanodes was estimated via Kublenka-Munk (K-M) functions, and the data are presented in Table 1. The assessed optical band gaps decreased in the order TaON > ZnTaO_2_N > CoPi/ZnTaO_2_N > LaTa_0.3_Nb_0.7_O_2_N > LaNbO_2_N (Table 1), which is consistent with a similar effect for perovskite tantalum oxides [42].

### 2.2. Morphological Characteristics of ZnTaO_2_N Photo-Anodes

**Figure 3:**
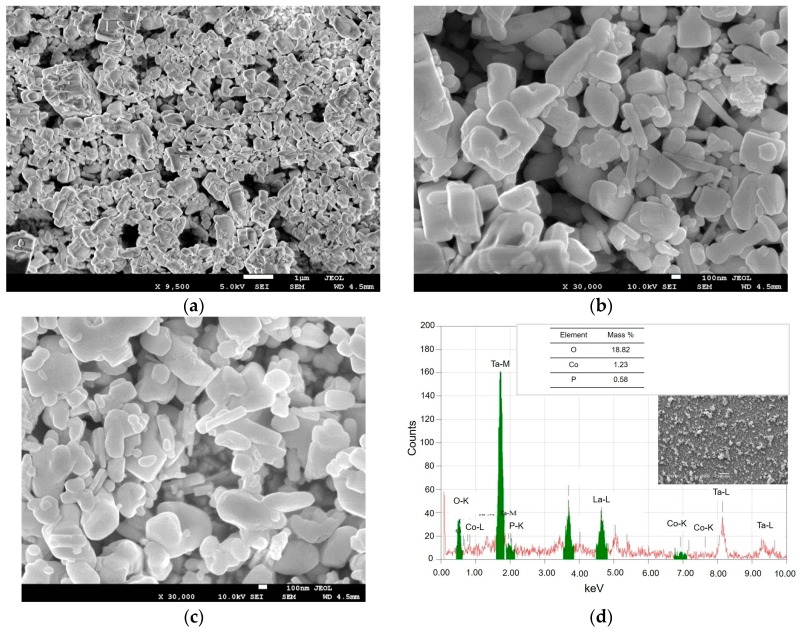
Scanning electron microscopy (SEM) images of (**a**) ZnTaO_2_N; (**b**) higher magnification of ZnTaO_2_N; (**c**) CoPi/ZnTaO_2_N photo-anodes prepared via electrophoretic and photo-deposition deposition onto ITO; (**d**) energy-dispersive X-Ray analysis (EDX) spectrum of CoPi/ZnTaO_2_N.

Figure 3 displays SEM images of the surface morphology of the ZnTaO_2_N (Figure 3a,b) and CoPi/ZnTaO_2_N (Figure 3c) photoanodes. These display irregular shapes for both ZnTaO_2_N and CoPi/ZnTaO_2_N materials with the presence of some voids between the particles. The particle sizes generally range from 30 to 200 nm. The ZnTaO_2_N particles had clearer crystal edges and faces, indicating a superior crystallinity of these anodes. This is likely to favor the process of photohole generation, which can reach the active reaction sites at the boundary between the electrode/electrolyte [30]. However, the presence of large quantities of grain boundaries and loose inter-particle connections may lead to the incoherence of electron transport between the particles. The SEM image in Figure 3c for ZnTaO_2_N covered with CoPi clearly reveals that the loaded CoPi layer does not alter the morphology of ZnTaO_2_N particles. In order to prove the deposition of the CoPi layer at the ZnTaO_2_N surface, we examined the structure using energy-dispersive X-Ray analysis (EDX), and the results are shown in Figure 3d. The EDX characterization confirms the existence of Ta, Co, and P elements in the photoanodes at 46.25, 1.23, and 0.58 wt %, respectively.

### 2.3. XPS Investigation of the CoPi/ZnTaO_2_N Photoanodes

**Figure 4:**
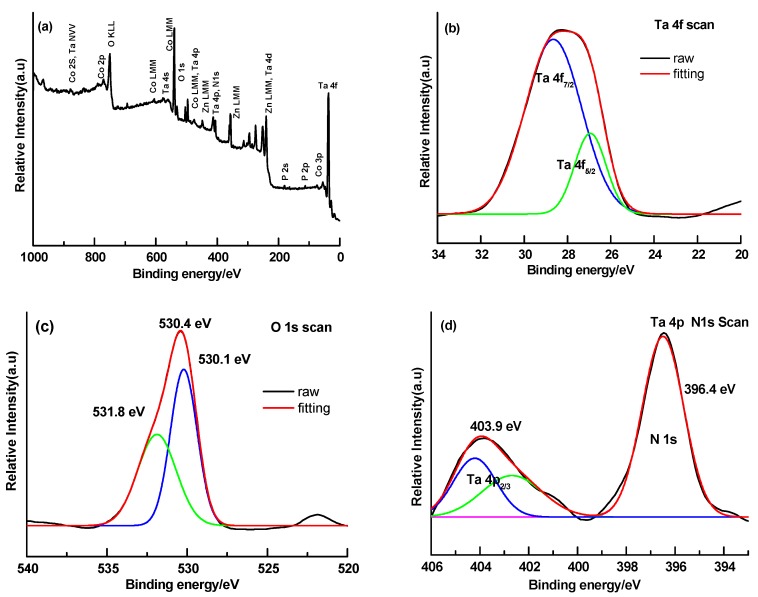
X-ray photoelectron spectroscopy (XPS) analysis of the CoPi/ZnTaO_2_N microsphere: (**a**) wide survey spectra scan for sample; narrow scan for (**b**) Ta element; (**c**) O element; (**d**) Ta and N element.

Figure 4 shows the existence of Co, P, Zn, Ta, O, and N in the wide-scan X-ray photoelectron spectroscopy (XPS) spectrum of CoPi/ZnTaO_2_N photo-anodes. The molar ratio of Co and P in CoPi/ZnTaO_2_N was estimated to be 1:2.2. The major peaks of binding energies at 26.8 and 28.5 eV related to the spin-orbit separation of the Ta 4f_5/2_ and Ta 4f_7/2_ ingredients, respectively (Figure 4b), demonstrating the development of the Ta^5+^ [43]. The two dissimilar binding energies of the O element can be allocated to the characteristics of the Ta-O bond (530.1 eV) and oxygen in carbonate species or hydroxyl groups (531.8 eV) (Figure 4c) [44]. The major peaks for Ta 4P_2/3_ and N 1s in Figure 4d indicate that the N 1s region centered at 396.4 eV is associated with the binding energy of about 403.5 eV for Ta 4P_2/3_, further confirming the creation of various Ta-N bonds [44].

### 2.4. Photoelectrochemical (PEC) Properties of the ZnTaO_2_N Photoanodes

**Figure 5:**
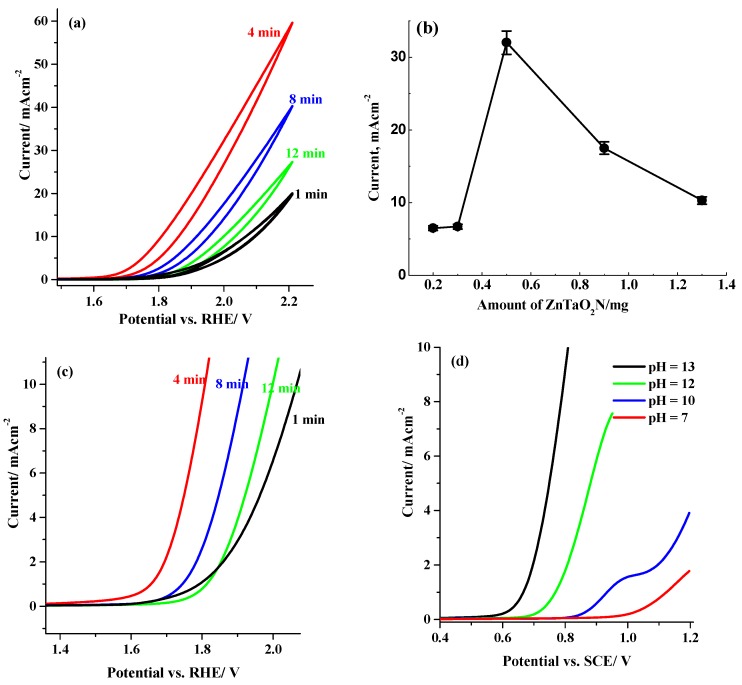
(**a**) Cyclic voltammetry (CV) at 50 mV·s^−1^ in 1.0 M Na_2_SO_4_ at pH 13 for photocurrent response for various amounts of ZnTaO_2_N loaded on ITO; (**b**) plot for photocurrent measured at 2.0 V vs. RHE and amount of loaded ZnTaO_2_N deposited by electrophoretic deposition (EPD) onto ITO; (**c**) CV in (**a**) zoomed in current less than 10 mA·cm^−2^; (**d**) Linear sweep voltammetry (LSV) recorded at various pH of electrolyte solution in the presence of 1.0 M Na_2_SO_4_ solution.

The PEC properties of ZnTaO_2_N photoanodes were examined by performing cyclic voltammetry (CV) and chronoamperometric (CA) measurements in an H-shaped cell. Figure 5a shows the CV measurements logged at 50 mV·s^−1^ in 1.0-M Na_2_SO_4_ for ZnTaO_2_N/ITO photoanodes under AM 1.5 G simulated sunlight at various thicknesses of the film prepared by varying the EPD deposition time duration. The results show that the current density of the photoanode changes dramatically as the deposition time increases. As shown in Figure 5b, the maximum current density (measured at 2.0 V vs. RHE) was observed around 0.5 ± 0.05 mg of loaded ZnTaO_2_N on ITO substrate (equivalent to deposition for 4.0 min and a thickness of 2.0 μm), and at higher loading (>0.5 mg), the photoanode current density significantly decreased. This can be clarified by the catalytic water oxidation reaction at the CoPi/electrolyte interface, and when CoPi loading at higher loading (>0.5 mg), the photoholes need to be transferred in amid numerous CoPi molecules and CoPi/electrolyte interface, which affects the sluggish kinetics of the hole transfer, and subsequently, a smaller photocurrent is detected [32]. Moreover, as shown in Figure 5c (taken from Figure 5a), at a ZnTaO_2_N loading of around 0.5 ± 0.05 mg, the onset potential of water oxidation markedly shifted to a less positive value, indicating a more favorable process. To understand the effect of pH on the PEC behavior of the ZnTaO_2_N photoanode, Figure 5d shows the linear sweep voltammetry (LSV) plot for ZnTaO_2_N photoanode at different pH in 1.0-M Na_2_SO_4_ solution. It is evidenced that the current density of water oxidation was significantly enhanced at alkaline pH of 13, which is almost eight times superior than that at pH of 12. Additionally, the onset potential of water oxidation was shifted 120 mV more cathodic compared with the value obtained at pH of 12.

**Figure 6:**
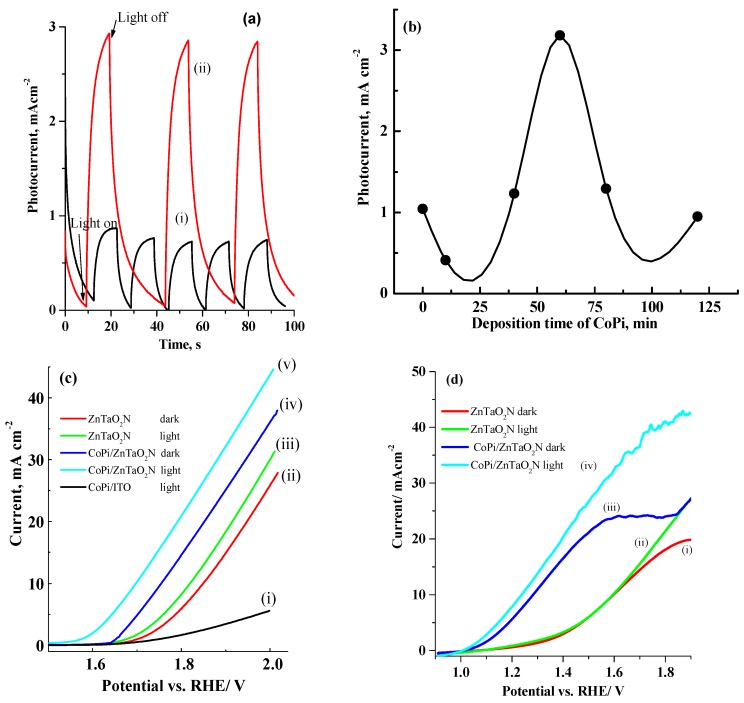
(**a**) Chronoamperometry diagram at 1.7 V vs. RHE during pulsing of light for 10 s in 1.0-M Na_2_SO_4_ solution (pH 13) for (i) ZnTaO_2_N and (ii) CoPi/ZnTaO_2_N photoanode, photodeposition time = 60 min; (**b**) plot for the photocurrent vs. deposition time of CoPi deposition on ZnTaO_2_N photoanode; (**c**) LSV at 50 mV·s^−1^ in dark and under 1.5 AM light illumination for (i) CoPi/ITO in light, (ii) ZnTaO_2_N in dark, (iii) ZnTaO_2_N in light, (iv) CoPi/ZnTaO_2_N in dark, and (v) CoPi/ZnTaO_2_N in light; photo-anodes in 1.0-M Na_2_SO_4_ solution (pH 13) and (**d**) LSV spectra of ZnTaO_2_N-based photoanodes in the presence of H_2_O_2_ (i) ZnTaO_2_N in dark, (ii) ZnTaO_2_N in light, (iii) CoPi/ZnTaO_2_N in dark, and (iv) CoPi/ZnTaO_2_N in light.

To further improve the PEC behavior of the ZnTaO_2_N photocatalyst for water oxidation reaction, the CoPi co-catalyst (OEC) was incorporated into the photoanodes using the photodeposition PD method [32,45]. Initially, the amount of CoPi loaded onto the ZnTaO_2_N film was optimized by varying the PD duration, followed by measurement of the photocurrent response using chronoamperometry at 1.7 V vs. RHE in 1.0-M Na_2_SO_4_ solution. Figure 6a shows the current time transients with a 10 s light pulse (1.5 AM) for a ZnTaO_2_N anode before and after the photodeposition of CoPi for a deposition time of 60 min. For clarity, the background current in the dark was subtracted for both photoanodes. Once the light pulse was applied, the current of both photoanodes increased significantly and the photocurrent density obtained at the ZnTaO_2_N anode was about 0.75 mA·cm^−2^, while the CoPi/ZnTaO_2_N photoanode displayed a much superior photocurrent density of 2.9 mA·cm^−2^ under the similar conditions. Clearly, the photocurrent obtained at CoPi/ZnTaO_2_N is more than three times higher than in the case of the ZnTaO_2_N electrode, which indicates that the separation and collection processes of the photogenerated electron/hole pairs are more efficient at CoPi/ZnTaO_2_N than at a simple ZnTaO_2_N photoanode.

Figure 6b shows the relationship between the CoPi PD time and the photocurrent obtained for the CoPi/ZnTaO_2_N anode at 1.7 V vs. RHE. Clearly, using our present PD methodology, the optimum period for CoPi photo-deposition on ZnTaO_2_N photo-anodes was found to be around 60 min. The PEC characteristics were compared for both ZnTaO_2_N and CoPi/ZnTaO_2_N anodes to examine the effect of the photodeposited CoPi co-catalyst on the photocurrent response of the ZnTaO_2_N photoanode. Figure 6c shows the LSV at 50 mV·s^−1^ in the dark and under 1.5 AM light illumination for CoPi/ZnTaO_2_N and ZnTaO_2_N photoanodes in 1.0-M Na_2_SO_4_ solution (pH 13). In comparison, the LSV of the control anode made by the PD of the CoPi catalyst directly onto the ITO substrate is also shown in Figure 6c (i). The LSV for the control anode of CoPi/ITO (i) shows a small level of the oxygen evolution current under light illumination. The CoPi/ZnTaO_2_N photoanode clearly shows negative potential shifts of about 120 mV in the onset potential of oxygen evolution, and an increase in the photocurrent of more than 2.3 mA·cm^−2^ at 1.23 V vs. RHE relative to its parent ZnTaO_2_N photoanode. For the CoPi/ZnTaO_2_N anode, the photocurrent was enhanced by a factor of three compared with the parent ZnTaO_2_N electrode. It is clear that the magnitude of the CoPi loading is influenced by the photoanode morphology, time, and the employed deposition procedure. A larger amount of CoPi deposited on ZnTaO_2_N (>60 min) results in the suppression of the photocurrent, as revealed in Figure 6b. This phenomenon can be explained by considering that the water catalytic oxidation reaction takes place at the CoPi/electrolyte interface. For a thick layer of CoPi, the photoholes have to transfer through a thick CoPi layer to reach the interface between the CoPi/electrolyte; consequently, the hole migration becomes very slow, and a decreased photocurrent is observed. On the contrary, for a thinner CoPi layer, the cobalt ions link more directly to the ZnTaO_2_N surface, and rapidly acquire the photoholes in the water oxidation reaction [46,47].

Hydrogen peroxide (H_2_O_2_) was introduced to the electrolyte (0.1 M) as an electron donor to evaluate the maximum photocurrent that might be acquired while injecting a photoexcited holes from the anode surface to the electrolyte solution was perfectly facilitated. It is believed that H_2_O_2_ captures the photoexcited holes efficiently without substantial recombination [27]. To observe these behaviors on the prepared photoanodes, LSV plots of CoPi/ZnTaO_2_N were logged in the presence and absence of H_2_O_2_ and the results are presented in Figure 6d. The LSV of ZnTaO_2_N and CoPi/ZnTaO_2_N photoanodes indicate that the photocurrent at applied potential values of more negative than 1.7 V vs. RHE was improved considerably in the presence of H_2_O_2_. This implies that the redox active Co species may be quickly oxidized at a lower applied potential (<1.4 V vs. RHE), while water could not. This results coincides with the results attained for CoPi/La(Ta,Nb)O_2_N photoelectrodes [32]. These PEC performances show that the CoPi/ZnTaO_2_N photoelectrodes, photocurrent at <1.4 V vs. RHE was comparable, irrespective of the introduction of H_2_O_2_, signifying that the CoPi had improved the surface redox reactions effectively.

### 2.5. Band Positions of the ZnTaO_2_N Photoanodes

As previously discussed, the band-edge position at the electrolyte and the carrier mobility in the semiconductor photocatalyst are both significant factors for the characterization of the PEC performance of the photoanodes. The Mott–Schottky (MS) method was performed and the Nyquist plot was obtained to determine the characteristics of ZnTaO_2_N and CoPi/ZnTaO_2_N electrodes in 1-M Na_2_SO_4_ (pH 13) at a frequency of 100 Hz. The valence-band and conduction-band potentials of TaON, LaTaO_2_N, and ZnTaO_2_N were calculated using the Butler and Ginley method, and the values are précised in Table S1.

**Figure 7:**
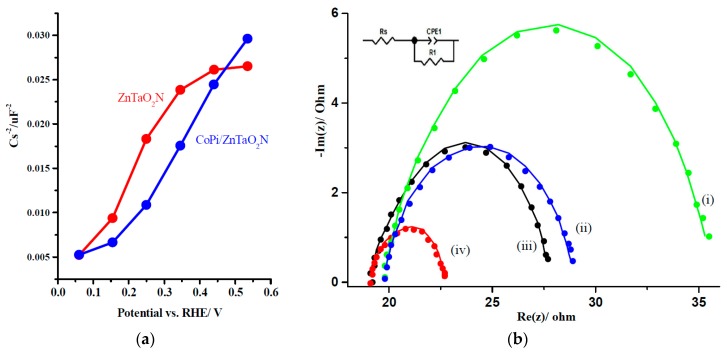
(**a**) Mott–Schottky plots for ZnTaO_2_N and CoPi/ZnTaO_2_N photo-anodes; (**b**) Nyquist plot for the photoanodes of (i) ZnTaO_2_N in dark, (ii) ZnTaO_2_N in light, (iii) CoPi/ZnTaO_2_N in dark, and (iv) CoPi/ZnTaO_2_N in light; illumination conditions are 1.5 AM and in 1.0-M Na_2_SO_4_ at pH = 13.

The conduction-band edge (E_CB_) for ZnTaO_2_N was obtained at 0.32 V vs. SCE by employing the method proposed by Butler and Ginley [48] using the optical BG from the K-M spectra. As presented in Figure 7a, the intercept on the potential axis of the ZnTaO_2_N photoanode reveals a flat band potential (E_FB_) at 0.14 V, while the intercept of the CoPi/ZnTaO_2_N photoanodes displays an E_FB_ at 0.08 V vs. RHE at pH = 13. It is clear that CoPi/ZnTaO_2_N photoanodes have less positive E_FB_ values than the parent photoanodes. The addition of the CoPi co-catalyst as a hole acceptor promotes the charge-separation reaction, which leads to the enhancement of the photocurrent during the water oxidation reaction. In addition, the CoPi co-catalyst can cause the oxygen evolution reaction to occur at lower oxidation potential by altering the reaction pathway [39]. The CoPi layer on the photoanode has to be thin enough to complement the fast transfer of the photogenerated hole from the ZnTaO_2_N photoanode to the water oxidation reaction, which relieves the charge accumulation at the electrode/electrolyte interface.

Figure 7b shows Nyquist plots of the ZnTaO_2_N and CoPi/ZnTaO_2_N photo-anodes acquired in the light and in the dark. The equivalent circuit is shown in the inset of Figure 7b and the fitting results of the photoanodes are summarized in Table 2. The ZnTaO_2_N photo-anodes were perfect fitted to a RC circuit model which grasps a resistor and a RC circuit. RC circuit can be allocated to ZnTaO_2_N/electrolyte interface. For the CoPi/ZnTaO_2_N photo-anode, capacitances increased, whereas the charge transfer resistances decreased, signifying that CoPi-loading sustained charge separation at the bulk photo-anode and, as a consequence, enriched PEC water oxidation ability. CoPi loading on photoanodes advances the PEC behaviour by endorsing the charge separation and water oxidation phenomenon on the ZnTaO_2_N photo-anodes surface which is very consistent with the behavior of CoPi/LaTaNbO_2_N [32], CoPi/TiO_2_ [39] and CoPi/BiVO_4_ structures [47].

### 2.6. Quantification of Dioxygen Evolution during Photoactivation

**Figure 8:**
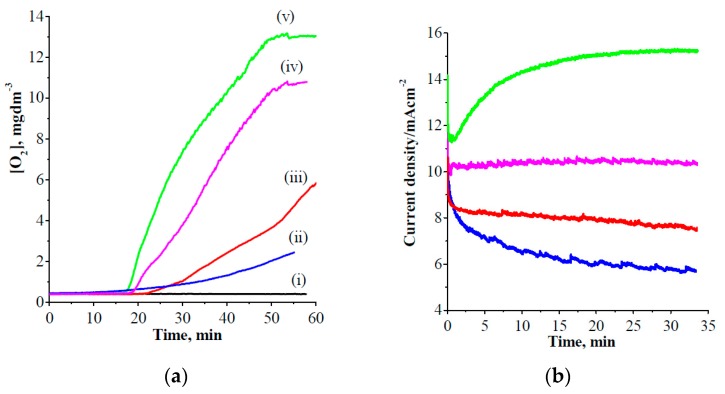
(**a**) Oxygen evolution and (**b**) its corresponding chronoamperometric measurements in a two-electrode setup in 1.0-M aqueous sulphate solution (pH = 13) during prolonged irradiation by visible light (λ > 420 nm), (i) ITO blank, (ii) ZnTaO_2_N dark, (iii) CoPi/ZnTaO_2_N dark, (iv) ZnTaO_2_N light, and (v) CoPi/ZnTaO_2_N light; the working electrode was biased at 1.7 V vs. RHE.

The irradiation of ZnTaO_2_N and CoPi/ZnTaO_2_N photoanodes with visible-light photons eventually increases the evolution of dioxygen. Figure 8a shows the oxygen-evolution measurements obtained at 1.7 V vs. RHE using an oxygen sensor oxysense system. The corresponding photocurrent responses were obtained and the results are presented in Figure 8b. As anticipated, under similar conditions, the CoPi/ZnTaO_2_N photoanodes and their parent photoanodes with visible-light photons (λ > 420 nm) caused an increase in the dioxygen evolution. To obtain an estimate, the precise oxygen concentration was logged before and after PEC reactions. Figure 8a confirms that by switching on the photoelectrolysis, the concentration of oxygen starts to increase linearly with time, and no substantial quantity of oxygen was observed before (<15 min) or after (>45 min) the photoelectrolysis was turned off. The CoPi/ZnTaO_2_N photoanodes showed increased oxygen generation relative to parent ZnTaO_2_N, which is demonstrated by its oxygen-evolution rate. The oxygen-evolution rate is faster in the case of CoPi-coated ZnTaO_2_N compared with the parent photoanode (Figure 8a). Conversely, it is evident that the incorporation of CoPi as a co-catalyst enhanced the photocurrent response and stability under continued illumination, as shown in Figure 8b. The durability of the CoPi/ZnTaO_2_N photoanodes was examined, and it displayed in Figure S1. The chronoamperometric results indicate that the photoanode remains stable until 180 min, after which small changes are observed. The stability of the CoPi/ZnTaO_2_N photoanodes under light irradiation was investigated by relating their absorption spectrum before and after visible light irradiation, signifying that the photoanodes are stable under visible-light illumination (Figure S2). A more effective coupling of photoholes to reaction sites of the water-oxidation reaction can be anticipated, as discussed earlier, which validates the enhancement of oxygen-evolution performance as well as the stability of CoPi-loaded ZnTaO_2_N photoelectrodes.

## 3. Experimental

### 3.1. Preparation of the ZnTaO_2_N Catalyst

The ZnTaO_2_N powder was fabricated via conventional solid-state reaction described in a previous work [49]. In the synthesis described here, stoichiometric quantities of ZnCO_3_ and Ta_2_O_5_ (Aldrich, 99.9%, St. Louis, MO, USA) were well ground together in acetone in the presence of KCl (50% total weight), which is used as a mineralizer. The resulting mixture was heated at 850 °C under an ammonia flow (Air Products Electronic Grade, Riyadh, KSA) for 20 h at a flow rate of 7 dm^3^·h^−1^, and it was then permitted to cool to normal temperature under ammonia atmosphere. The mineralizer was leached from the products using excess de-ionized water, and the residual product was dried overnight at 80 °C. The TaON was prepared following similar procedure by heating pure Ta_2_O_5_ (Aldrich, 99.9%) at 850 °C under flowing ammonia for 18 h at a flow rate of 7 dm^3^·h^−1^.

### 3.2. Fabrication of the ZnTaO_2_N Photoanodes

ZnTaO_2_N film photoanodes were fabricated using the EPD process, as described in our previous work [32]. For instance, the ZnTaO_2_N (15 mg) and 3-mg iodine (Alfa-Aesar, Karlsruhe, Germany) powders were kept ultrasonically discrete in acetone (15 mL) to obtain a uniform suspension. The ITO substrates (174 nm, 1.0 × 1.0 cm, Asahi glass Co., Ltd., Tokyo, Japan) were submerged and held parallel at about 1.0 cm apart from each other in the solution. A +10 V bias was then applied to the two electrodes for 2 min using a potentiostat (Bio-Logic SAS, VSP 0478, Seyssinet-Pariset, France). This deposition process was repeated twice, and the electrode was then dried and annealed at 450 °C under a flow of N_2_ gas at a rate of 500 mL/min for 1 h. This process resulted in the development of a ZnTaO_2_N layer (in total about 0.5 mg) with a comparatively uniform thickness of about 2 μm, as monitored using a surface profilometer. The cobalt phosphate (CoPi) co-catalyst layer was deposited on ZnTaO_2_N photoanodes using the PD method, as described in the literature [32]. CoPi/ZnTaO_2_N electrodes with various CoPi depositions were fabricated by altering the PD time (indicated as CoPi/ZnTaO_2_N). Characterization and XRD measurements were carried out using a MiniFlex 600 (Rigaku, CuKα, 40 kV, 15 mA, Tokyo, Japan). The photoanodes were further characterized using an ultraviolet visible (UV-Vis) spectrophotometer (Shimadzu UV-2600, Tokyo, Japan) and EDAX (JED-2200 series, Tokyo, Japan). Electrochemical impedance spectroscopy (EIS) analysis was performed using a biologic (VSP-0478) potentiostat electrochemical workstation. A solar simulator (Asahi spectra, MAX 303, Torrance, CA, USA) provided visible-light irradiation to the fabricated photoanodes.

### 3.3. Photoelectrochemical Characterization

The PEC properties of the ZnTaO_2_N and CoPi/ZnTaO_2_N photoanodes were obtained in H-shaped two-compartment glass cells with a 2-cm diameter quartz window. The fabricated ZnTaO_2_N-based photoanode film was taken as a working electrode (WE, 0.25 cm^2^), the saturated calomel electrode as a reference electrode, and Pt foil was served as the counter electrode in the electrolyte solution containing 1-M Na_2_SO_4_, and the pH of the electrolyte solution was modified to 13 with the addition of KOH. Photoelectrochemical oxygen evolution was monitored by an oxygen analyzer (Oxysense Inc., Dallas, TX, USA, 300/5000 series). The compartment cell was sealed and argon gas was used for purging. Prior to the experiment, the electrolyte solution was bubbled with argon for 1 h, and the atmosphere above the electrolyte was maintained as argon throughout the measurements. Before starting the electrolysis, the cell setup was purged for 20 min to attain the equilibrium.

## 4. Conclusions

A photocatalyst of ZnTaO_2_N was initially synthesized via conventional solid state reaction and then photoanodes were fabricated by an electrophoretic deposition method into ITO. We studied the co-catalytic effect of a photo-assisted water oxidation reaction by photodeposition of CoPi oxygen electrocatalyst onto the ZnTaO_2_N photoanodes. An electrochemical investigation revealed that the PEC performance of ZnTaO_2_N was significantly enhanced after CoPi deposition and a 2.3 mA·cm^−2^ photocurrent density was reached at 1.23 V vs. RHE in a sulfate medium. Additionally, CoPi-loading assisted the PEC performance of ZnTaO_2_N film by reducing the charge recombination process and stabilizing the photo-anode performance for the oxygen evolution reaction under visible light illumination.

## Figures and Tables

**Table 1:** Structural properties and the band gap of various oxynitride based photoanodes.

Electrophoretic Deposited Oxynitrides on ITO	Particle Size (nm) ^[a]^	Band Gap *E_g_* (eV) ^[b]^
TaON	29.5	2.95
ZnTaO_2_N	22.7	2.75
CoPi/ZnTaO_2_N	22.4	2.7, 2.47
LaTa_0.3_Nb_0.7_O_2_N [32]	18	1.84
LaNbO_2_N	40	1.65

^[a]^ Obtained using Scherer equation; ^[b]^ Determined from the diffuse reflectance spectroscopy (DRS) analysis via K-M function.

**Table 2:** Nyquist plot of equivalent circuit-model parameters obtained from the electrochemical impedance spectra of photoanodes.

Photoanode		*R*s/Ω	*R*ct/Ω	CPE1 (F)
ZnTaO_2_N	Dark	22.17	33.55	6.763 × 10^−5^
	Light	22.17	27.23	5.714 × 10^−5^
CoPi/ZnTaO_2_N	Dark	19.17	25.81	1.15 × 10^−4^
	Light	19.17	22.46	2.303 × 10^−5^

## Supplementary Materials

The following are available online at www.mdpi.com/2079-4991/8/1/48/s1, Table S1: Photophysical and structural properties of TaO_2_N based photoandodes, Figure S1: Chronoamperometric measurements in two-electrode setup in 1 M aqueous sulfate solution (pH = 13), Figure S2: Absorption spectra of CoPi/ZnTaO_2_N photoanodes before and after visible-light irradiation for 10 h.
