# Supplementary material for: Zinc Tantalum Oxynitride (ZnTaO2N) Photoanode Modified with Cobalt Phosphate Layers for the Photoelectrochemical Oxidation of Alkali Water
Source: Nanomaterials (Basel). 2018 Jan 18;8(1):48. doi: 10.3390/nano8010048 (PMC5791135; doi:10.3390/nano8010048)
Supplement: Supplementary file 1 [file nanomaterials-08-00048-s001.pdf]

nanomaterials

Supporting Information

Zinc Tantalum Oxynitride ($ZnTaO_2N$) Photoanode Modified with Cobalt Phosphate Layers for the Photoelectrochemical Oxidation of Alkali Water

Prabhakarn Arunachalam [1,*], Maged N. Shaddad [1], Mohamed A. Ghanem [1], Abdullah M. Al-Mayouf [1] and Mark T. Weller [2]

1 Electrochemistry Research Group, Chemistry Department, College of Science, King Saud University, Riyadh 11451, Saudi Arabia; mshadad@ksu.edu.sa (M.N.S.); mghanem@ksu.edu.sa (M.A.G.); amayouf@ksu.edu.sa (A.M.A-M.)

2 Department of Chemistry, University of Bath, Bath BA2 7AY, UK; m.t.weller@bath.ac.uk

* Correspondence: parunachalam@ksu.edu.sa; prabhuchemist@hotmail.com; Tel.: +00-966-114696026

Received: 18 November 2017; Accepted: 28 December 2017; Published: date

Table S1. Photophysical and structural properties of TaO_2N based photoandodes.

Sample	Thickness (μm)[a]	χ	E_{cb}[c]	E_{vb}[c]	Band gap E_g (eV)[d]
TaON	0.4	6.3	0.325	3.275	2.95
$LaTaO_2N$	0.3	5.9	0.110	2.690	2.58
$ZnTaO_2N$	0.4	6.2	0.359	3.100	2.75

[a] Thickness of photoanodes measured by the profilometer. [b] Obtained from experimental analysis of Mott-Schotky plot. [c] Obtained by Butler and Ginley method by considering the electronegativity of the particles. [d] determined from DRS analysis using Kublenka-Munk function.

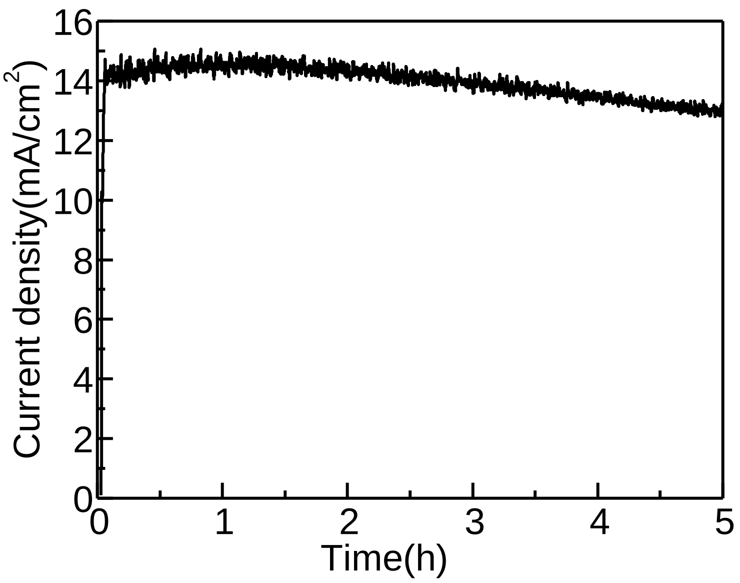

Figure S1. Chronoamperometric measurements in two-electrode setup in 1 M aqueous sulfate solution (pH = 13), during prolonged irradiation of visible light (λ > 420 nm).

nanomaterials

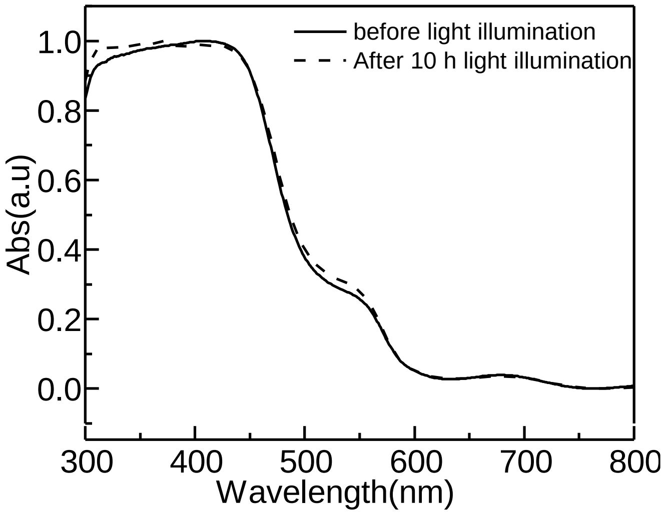

Figure S2. Absorption spectra of CoPi/$ZnTaO_2N$ photoanodes before and after visible-light irradiation for 10 h. No changes in the absorption spectra of the nanoparticles before and after irradiation.

© 2017 by the authors. Submitted for possible open access publication under the terms and conditions of the Creative Commons Attribution (CC BY) license (http://creativecommons.org/licenses/by/4.0/).
